# Case report and literature review: autonomous robotic system assisted palatal implantation at an anterior teeth site compromised by periapical cyst

**DOI:** 10.3389/fmed.2024.1335043

**Published:** 2024-01-15

**Authors:** Xiuyu Liu, Huixin Lv, Meiqing Chen, Siyu Chen, Kewen Jia, Sezhen Quni, Lu Zhang, Yanmin Zhou

**Affiliations:** ^1^Hospital of Stomatogy, Jilin University, Changchun, China; ^2^Department of Stomatology, China-Japan Union Hospital of Jilin University, Changchun, China

**Keywords:** immediate implant, aesthetic area, dynamic navigation, static navigation, case report

## Abstract

**Background:**

Immediate implant placement (IIP), which preserves gingival height and papilla shape while simultaneously accelerating the implant treatment period, has become a popular method due to its commendable clinical outcomes. Nonetheless, deploying immediate implants demands specific preconditions concerning the remaining alveolar bone. This poses a challenge to the accuracy of implant surgery.

**Case presentation:**

In this report, we present the case of a 60-year-old woman with a left upper anterior tooth crown dislodged for over a month. Cone beam computed tomography (CBCT) revealed the absence of a labial bone wall on tooth 22, a remaining 1 mm bone wall on the labial side of the root apex, and a 17.2 mm*8.9 mm*4.7 mm shadow in the periapical region of the root apices of teeth 21 and 22, with the narrowest width on the sagittal plane being approximately 5 mm. After the surgeon removed the cyst, they completed the subsequent implantation surgery using an autonomous robot in a challenging aesthetic area. This method circumvented the potential exposure of the screw thread on the labial implant surface, assured initial implant stability.

**Conclusion:**

Five months after the operation, the dental crown was restored. The implant remained stable, with yielding notable clinical results. To the best of our knowledge, this clinical case is the first to report the feasibility and precision of immediate implantation in anterior teeth site with periapical cyst removal, performed by an autonomous robotic surgical system. Autonomous robots exhibit exceptional accuracy by accurately controlling axial and angular errors. It can improve the accuracy of implant surgery, which may become a key technology for changing implant surgery. However, further clinical trials are still needed to provide a basis for the rapid development of robotic surgery field.

## 1 Introduction

The development of medical technology and biocompatibility materials has provided a variety of treatments for tooth defects caused by caries, trauma and tumors, but due to aesthetic requirements, leading to restoration of the anterior teeth remains a challenge. Immediate Implant Placement (IIP), which preserves gingival height and papilla shape while simultaneously accelerating the implant treatment period, has become a popular method due to its commendable clinical outcomes (1). A salient advantage of IIP is its ability to eliminate the bone healing stage post tooth extraction, thereby significantly abbreviating the implant treatment duration (2). Moreover, this approach facilitates the preservation of the soft tissue structure, culminating in superior aesthetic results (3).

Nonetheless, deploying immediate implants in aesthetic areas demands specific preconditions concerning the remaining alveolar bone. These include: a labial bone wall of at least 1 mm thickness; a thick gingival biotype; the absence of acute inflammation within the alveolar fossa; and sufficient bone mass in the root of alveolar fossa and palatal side to guarantee the initial stability of implant in its accurate three-dimensional position (4). As these teeth are primarily extracted due to acute or chronic inflammation, inflammation-induced bone defects can impede the initial stability of implant or result in thread exposure, thus complicating implant precision (5).

For the assurance of initial implant stability and long-term survival, maximal utilization of the remaining alveolar ridge is essential. When the buccal alveolar ridge is insufficient, palatal implant placement is proposed. This creates a 2 mm gap on the buccal side, and an augmentation osteotomy is performed to ensure the longevity of the implants (6, 7). Critical factors for a successful procedure include precise planning of the ideal implant position and accurate transfer of this planned position to the surgical site. Because free-hand manipulation is affected by many factors, including doctor’s experience, environment and patient’s cooperation, some scholars consider using guide plate to reduce human error. With the development of Science and Technology, robotic surgery has attracted much attention because of its high accuracy. (8, 9). The autonomous robotic system, pioneered by Professor Zhao, executes implant insertion as per preoperative design, with surgeons intervening when necessary (10). With the high accuracy of robot, the implant can be precisely placed at the preoperative design site in a critical bone defect while simultaneously avoiding implant thread exposure. To the best of our knowledge, this clinical case is the first to report the feasibility and precision of immediate implantation in an aesthetic area with periapical cyst removal, performed by an autonomous robotic surgical system, followed by a literature review.

## 2 Case presentation

**Figure 1:**
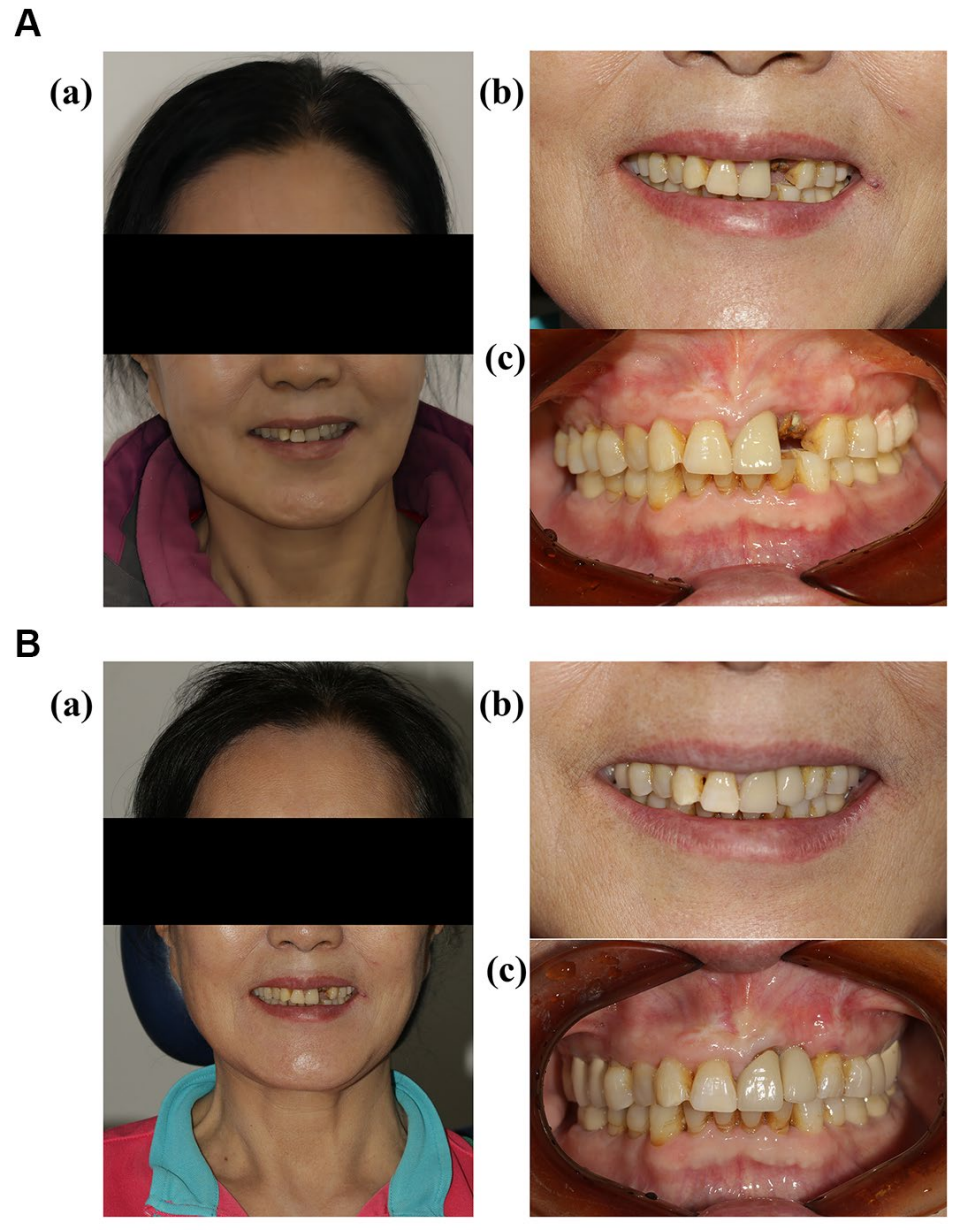
**(A)** (a) Frontal images of the patient before the operation; (b,c) intraoral images of the patient before the operation. **(B)** (a) Frontal images of the patient 5 months after the permanent restoration; (b,c) intraoral images of the patient 5 months after the permanent restoration.

**Figure 2:**
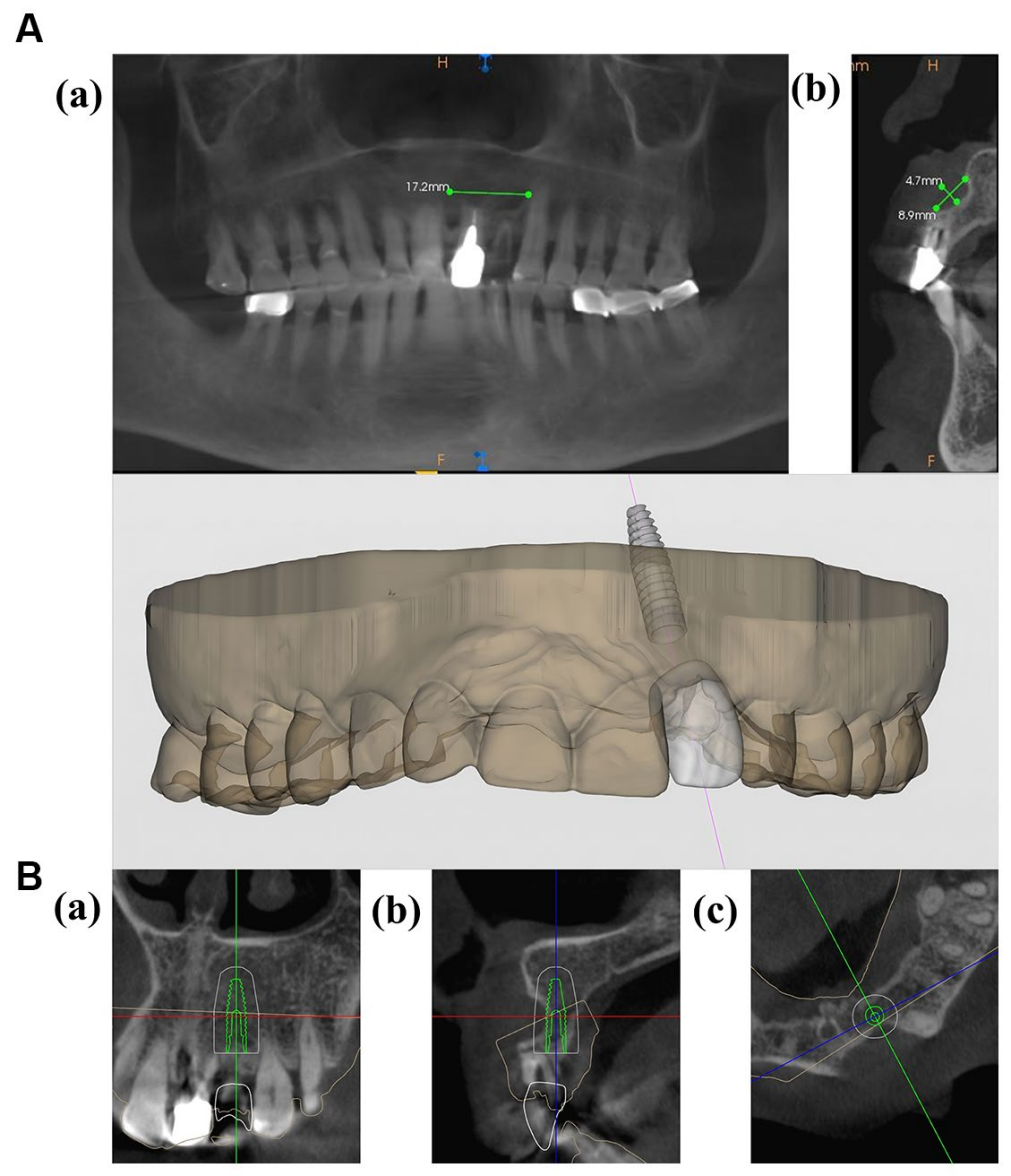
**(A)** (a,b) CBCT images of the patient before the operation. **(B,C)** Preoperative planning for implant placement.

The patient is a 60-year-old woman presenting with a left upper anterior tooth crown dislodged for over a month. The chief complaint suggested that an injury to her left upper anterior tooth a month prior, causing the original restoration to dislodge. Her dental history revealed a crown restoration on the upper anterior tooth a decade ago. Upon clinical examination, the following observations were made: a residual crown on tooth 22, normal gingival color and texture without redness, a thin gingival type of lip gingiva, a median laughing line, and a normal jaw position relationship. Tooth 21 had a post and a core crown (Figure 1A). Cone beam computed tomography (CBCT) revealed the absence of a labial bone wall on tooth 22, a remaining 1 mm bone wall on the labial side of the root apex, and a 17.2 mm*8.9 mm*4.7 mm shadow in the periapical region of the root apices of teeth 21 and 22, with the narrowest width on the sagittal plane being approximately 5 mm (Figure 2A). The diagnosis comprised a residual crown on tooth 22 and periapical cysts on teeth 21 and 22. The preoperative aesthetic risk assessment was moderate to low, despite the presence of apical labial bone defects and high aesthetic risk factors associated with adjacent teeth with prostheses (11).

A CBCT scan was conducted on the day of the patient arrived. The results and the virtual plan was then digitally transmitted to the robot (YaKeBot, DRS0605-FT250). The oral cavity of patient was scanned, and a three dimensional (3D) printer was used to create a positioning guide (Figures 2B,C).

**Figure 3:**
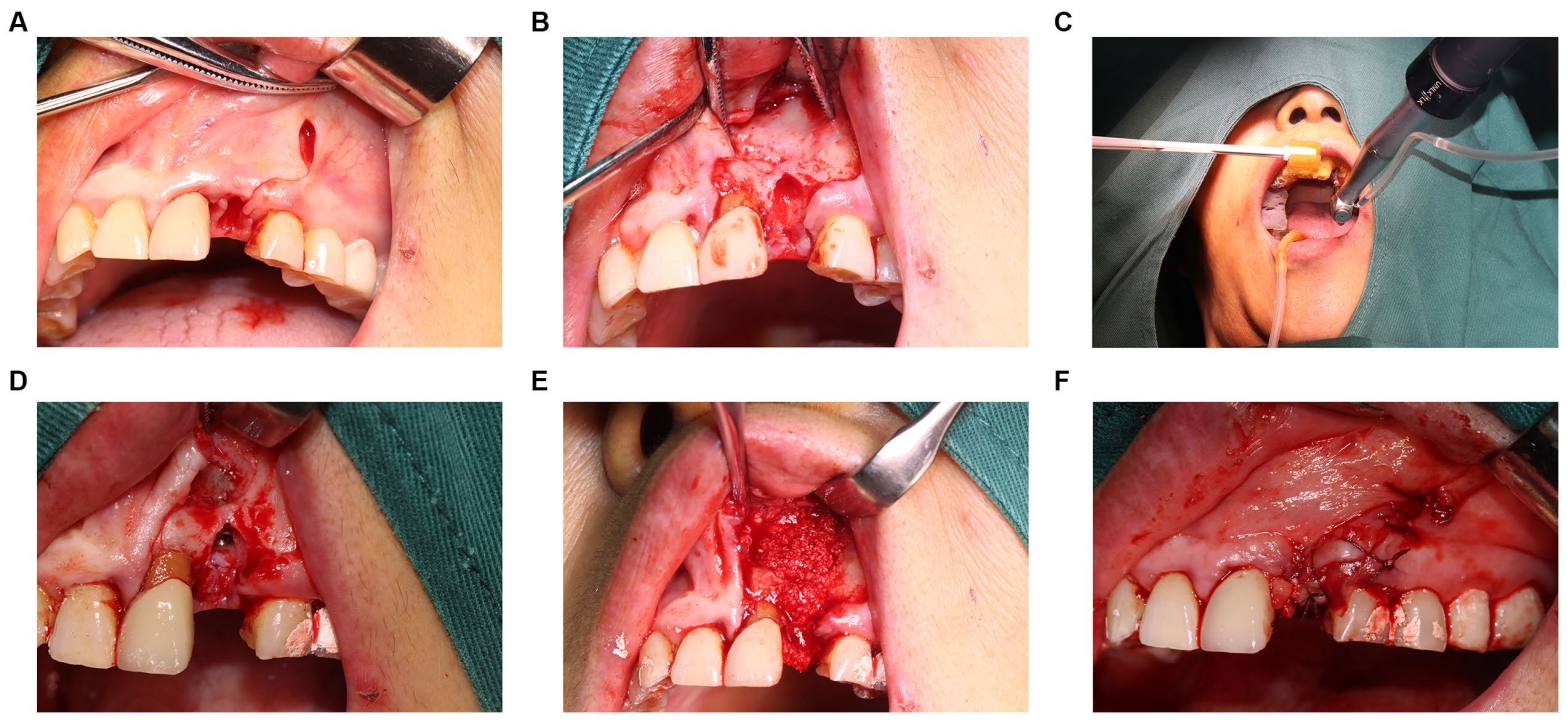
Intraoperative images. **(A,B)** Removal of cyst tissue and affected teeth. **(C)** The robot performs drilling and implant placement based on a pre designed path. **(D)** The implant has been placed in the alveolar bone. **(E)** Placement of bone powder to fill bone defects. **(F)** Tightly sutured wound.

**Figure 4:**
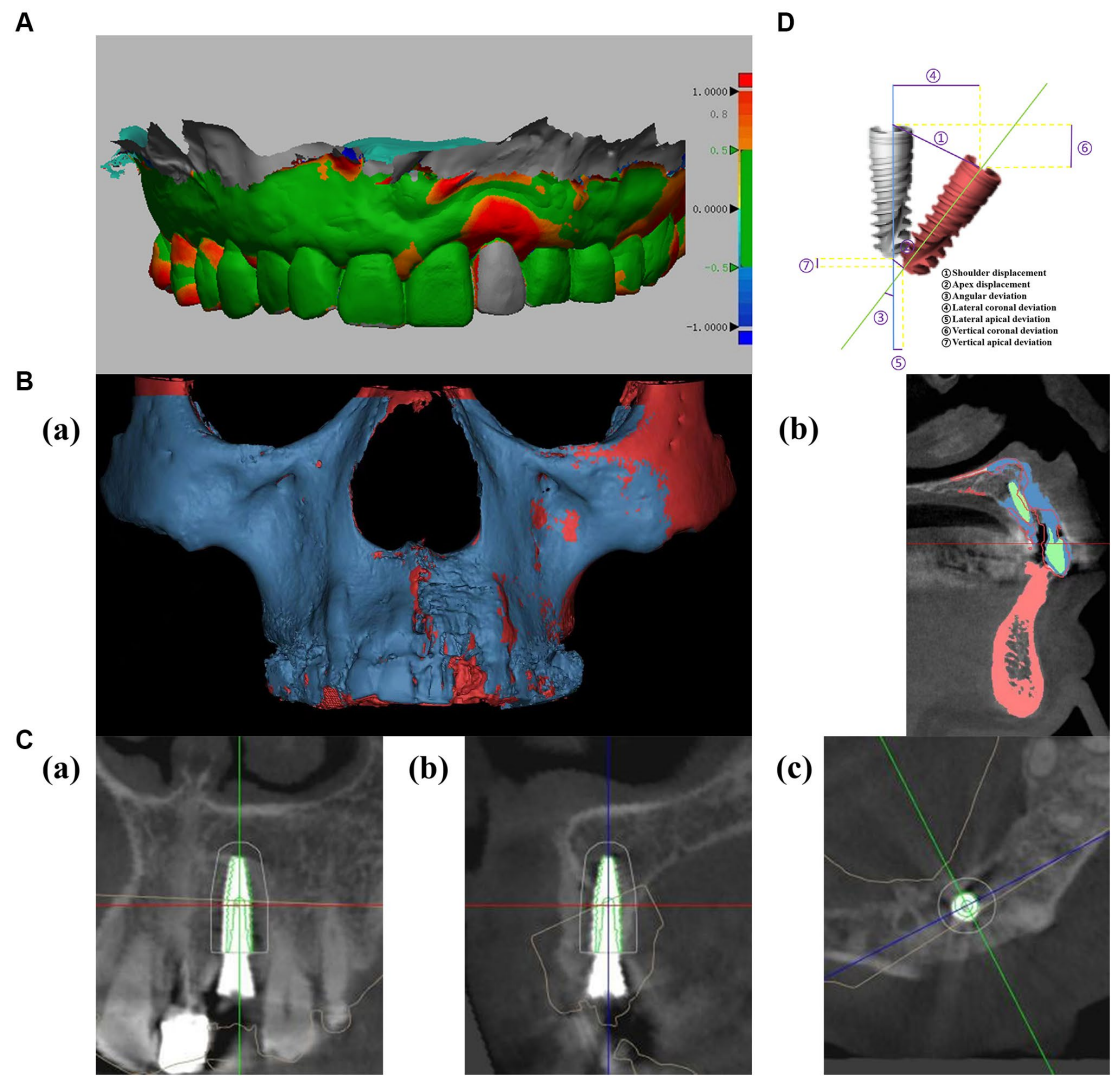
**(A)** Analysis of soft tissue recession using oral scanning data (soft tissue volumes were compared by comparing pre-and post-operative oral scan data, and color represented differences in gingival changes, which could be defined by the right scale). **(B)** Analysis of bone volume changes using CBCT (red represents preoperative bone tissue, blue represents postoperative bone tissue, and green represents implants and restorations). **(C)** Postoperative evaluation: comparing accuracy of preoperative planned implant position (green) with postoperative position. **(D)** Deviations of planned (gray) and actual position (red) of implant.

Before proceeding, a comprehensive treatment plan was presented to the patient, and written informed consent was obtained for the implantation procedure. Following local infiltration anesthesia, the tooth was extracted. Subsequent to the removal of the periapical cyst tissue via a flap technique, inflammatory soft tissue was meticulously cleared away. A temporary positioning guide plate was affixed to the maxillary dentition of patient for movement monitoring. Once the robotic tracking arm was calibrated, the surgeon, acting as the operator, released the robot arm, moving it to the surgical site for implant placement. A single implant (Straumann Bone Level Tapered Roxolid SLA, 3.3 mm*12 mm) was inserted intraoperatively with a torsional force of approximately 35 N cm, achieving satisfactory initial stability. Subsequent to the placement of bone powder in the labial bone defect, collagen and RPF membranes (Bio-Gide 13 mm*25 mm; Bio-Oss, 0.25 g, Switzerland) were placed, and a tension-free suture closed the wound (Figure 3). A CBCT scan was carried out immediately post-surgery to examine the implant placement. The entire procedure lasted for a total of 70 min. The calibration of the machine required 10 min, while the implantation executed by the robot took 20 min. The patient experienced no notable discomfort or adverse reactions. Implant discrepancies were evaluated by comparing the pre-designed STL files with the immediate post-operative CBCT (Figures 4C,D).

CBCT indicated that 22 labial bone plates exhibited satisfactory fullness, thereby assuring the function and aesthetic appeal of both soft and hard tissues in the implant area. Changes in the soft and hard tissues before the operation and after the operation were compared (Figures 4A,B). Resonance frequency analysis (PFA) was used to measure the stability of implants. PFA reflects the stiffness of bone-implant interface by implant stability quotient (ISQ) value. The (ISQ) was above 65, fulfilling the restoration standard (12). The final crown restoration was then completed by digitizing the casts (Figure 1B). The gingival color, shape, and texture recovered well, and the alveolar bone contours were full within 5 months post-implantation. The Pink Esthetics Score (PES) stood at 10 (13). CBCT and STL data were imported into Mimics Medical 21.0 and Geomagic Design X. This software was employed to calculate the bone volume increase to 455.4 cubic millimeters, while the soft tissue regression was measured at 1.94 mm.

## 3 Discussion

Immediate implantation condenses the treatment cycle and preserves existing soft and hard tissue, contributing to an enhanced aesthetic effect (14, 15). In our case, the labial bone of patient wall was missing due to a periapical cyst. According to the prevailing standard for immediate implantation, this condition is unsuitable for immediate implantation, and delayed implantation should be considered after the bone has healed. However, in our case, a robot-assisted palatal intraosseous implant was employed immediately following cyst removal. What’s even more remarkable, the clinical results demonstrate that the robotic surgery system efficiently utilizes the residual alveolar bone of patient, achieving substantial initial stability without exposing the implant surface. CBCT captured immediately after the operation revealed an error of about 0.4 mm between the implant site and the preoperative design. The crowns were restored 5 months after surgery, the mean volume of soft tissue change measured was 1.94 mm.

A previous research indicates that the vertical height of the labial alveolar bone of the implant significantly impacts the aesthetic appearance of the soft tissue and is closely tied to the placement of implant (16). Additionally, many studies have found that a labial alveolar bone thickness of at least 2 mm around implants is a positive factor for long-term soft tissue stability (17, 18). If bone mass is insufficient, delayed implantation, while more likely to ensure long-term implant stability, can result in severe alveolar-level resorption and soft-tissue retraction post-extraction, adversely influencing aesthetic prosthetics (15). Edith Groendijk in a prospective study, proposed fully utilizing palatal alveolar bone to achieve a minimum 2 mm gap with a buccal alveolar ridge as an effective method for addressing labial bone defects in the anterior region (6). In this case, the cyst caused a labial alveolar bone defect, but according to the CBCT, the average width of the palatal bone wall was 5 mm and the height of the bone was 19 mm. This could not only assure the stability of implant but also minimize the absorption of the labial bone wall and maintain the height and width of the labial bone. Therefore, we opted for palatal alveolar bone to carry out the implant operation and selected an implant with a diameter of 3.3 mm and a length of 12 mm to ensure complete implantation into the bone.

The degree of soft tissue alteration post immediate implantation within the aesthetic zone is a critical determinant of aesthetic outcomes. A mean soft tissue shrinkage of 0.27 ± 0.38 mm was observed in the mid-facial region following a 1–5 years follow-up study (19). The regression measured in this study amounted to 1.94 mm. According to previous literature, the causes of soft tissue retraction in this case can be attributed to the following: Firstly, the labial alveolar bone of the afflicted tooth exhibits a UU type bone defect. The research of Mizuno K established a notable positive correlation between alveolar bone defect severity and gingival recession (20). Additionally, the utilization of periodontal probes revealed the gums of patient to be of a thin gingival biotype (21), a significant risk factor for post-implant surgery gingival recession (22). The study of Nurit Bittner recorded a higher incidence of gingival recession in the thin phenotype (1.96 mm) compared to the thick phenotype (1.18 mm) (23). Secondly, alveolar bone defects following the removal of a periapical cyst also contribute to gingival retraction (24). Concomitantly, flap surgery is necessitated due to periapical cyst treatment and bone augmentation surgery, thereby escalating the risk of soft tissue retraction. The study of Filiep RaesLin compared the post-procedure outcomes of immediate implantation between flap surgery and flapless methods. Twenty-three patients underwent immediate implant placement while seven received conventional therapy. The immediate implant group exhibited a 43% reduction, compared to approximately 26% in the control group. Particularly, at the 40th week follow-up, the flapless technique demonstrated a significantly lesser decline than the flap method (25). Despite the use of robotic surgery, imperfect aesthetic outcomes were achieved, which was closely related to the aesthetic risk factors assessed preoperatively. This suggests that doctors should critically assess the risks and perform the surgery without compromising patient safety (26). In addition to surgical and anatomical factors, prostheses can also affect the shape of soft tissue. In fact, careful design is necessary because the shape, position, and color of the prosthesis can affect the shape and color of the soft tissue, which helps to simulate normal gingival contour. For patients with interdental papilla loss, the Oscar study suggests that an attempt to increase height can be made by changing the subcritical contour, which is closely related to soft tissue regeneration and plasticity (27).

In implant surgery, the three-dimensional positioning of the implant is critical for achieving long-term stability and optimal aesthetic results. Incorrect implant placement may induce marginal bone resorption and potentially infringe upon adjacent vital anatomical structures (28). In this case study, the autonomous robotic system employed optical sensors to ascertain the position relationship between the implant and the jaw, enabling accurate placement of the implant into the optimal location within the palatal bone. This took into account the depth and inclination, along with the biological, aesthetic, and functional considerations of the suprastructure, to support long-term aesthetic results and the health of peri-implant tissues. This system provides superior positioning accuracy compared to traditional navigation systems and can align the implant with the preoperative design. In this case, the deviation for shoulder displacement, apex displacement, and angular deviation were 0.42 mm, 0.42 mm, and 0.65°, respectively. These measurements indicate higher precision in comparison to reported errors from dynamic navigation (1.24 ± 0.39 mm, 1.58 ± 0.56 mm, 3.78° ± 1.84°), static navigation (0.87 ± 0.49 mm, 1.10 ± 0.53 mm, 2.41° ± 1.47°) and freehand methods (1.3 ± 0.7 mm, 2.2 ± 1.2 mm, 7.0° ± 7.0°) (29, 30). Current researches show that several factors affect the accuracy of digital navigation systems, including the precision of CBCT and oral scanning (or die removal), flap design, implant positioning, interference from cortical bone, stability of the guide plate, length of the drill bit and stem, compatibility between the guide rail and the drill bit, and the errors procedural of operator (31, 32). The accuracy of static navigation depends on the guide precision of plate. Factors that influence this include the cumulative errors from imaging examination, operation plan transfer, model construction, and guide plate fabrication. Moreover, the necessary sleeve tolerances (the space between the sleeve and the bit allowing for cooling water circulation and bit rotation) inherent in guide plate production can also impact the accuracy of implant procedures (33, 34). The research of Raico Gallardo suggests that tooth-supported guides demonstrate greater accuracy than mucosa-supported or bone-supported guides, possibly because tissue swelling from intraoperative local anesthesia can affect the positioning of guide plate (35). Dynamic navigation systems use sensors like cameras to locate the installed reference frame of patient and handheld device in real-time. These systems utilize CBCT data to calculate the relative spatial position between the patient and the drill bit, providing surgeons with real-time visual guidance during drilling (36). While dynamic navigation systems eliminate guide plate sleeve errors, they still require a guide plate system for location and calibration, tying their accuracy to guide plate manufacturing and placement. The accuracy of CBCT is crucial for scheme design and intraoperative guidance in dynamic navigation systems. Although CBCT delivers high accuracy in three-dimensional space, metal artifacts can affect image quality and precision (37). Akira Komuro indicated a discrepancy of 1.8–6.9% between CBCT measurements and actual values (38). Todorovic also noted that CBCT is not reliable for developing thin bone plates, which can impact the accuracy of dynamic navigation system (39). Robotic systems employ a real-time positioning system similar to dynamic navigation, which is also influenced by guide plate systems and CBCT errors. It is suggested that surgeons can minimize these errors by improving guide plate fabrication and placement precision, as well as CBCT accuracy. Notably, in addition to ensuring accuracy, the planting robot also uses mechanical sensors to prevent patient injury from the drill needle. The intraoperative robot can also adjust minor patient movements, ensuring accuracy and safety.

The autonomous robot not only demonstrated excellent accuracy, but also broke the technical sensitivity barrier of complex surgery. Immediate implantation in aesthetic areas is categorized as a type C operation, denoted as complex within the SAC classification [(S) simple, (A) advanced, (C) complex]. This is typically performed solely by implant surgeons with extensive experience, training, and education (4). Achieving complete bone wall coverage and initial implant stability can be challenging with free-hand immediate implantation. The application of robotic systems enables novice surgeons to independently perform complex procedures, fostering the growth of precision medicine technologies and significantly reducing the training period of surgeon. While static computer-assisted implant surgery (SCAIS) and dynamic computer-assisted implant surgery (DCAIS) provide higher accuracy than free-hand methods, they cannot entirely eliminate surgeon-related errors (40, 41). Neither static nor dynamic navigation can avoid direct intervention by the surgeon. Specifically, in dynamic navigation, the absence of a mechanical guidance device implies that the angle and position of drill and implant are entirely controlled by the surgeon (42). The study of Widmann suggests that a tremor and inaccurate hand perception could lead to an error of 0.25 mm and 0.5° (43). Few studies have analyzed the impact of clinical experience of a surgeon on the accuracy of static navigation. According to research by Van and Cassetta, implant placement errors in both experienced and inexperienced groups were primarily due to angular bias. Inaccurate placement of the guide plate was a significant factor contributing to implant inaccuracies (44). Dynamic navigation hinges on the hand-eye coordination of surgeon, which necessitates a learning period for proficiency in both navigation display data interpretation and implantation operations (45). However, the study of Gerardo Pellegrino posited that the accuracy of the dynamic navigation system was independent of the operator implant experience and familiarity with the dynamic navigation surgery, though noticeable differences were observed in drilling timing (46). The principal distinction between an autonomous robotic system and static and dynamic navigation lies in the utilization of robotic arms for implant procedures, with the surgeon functioning as the pilot of robot. The use of a robotic arm for drilling and implant placement effectively reduces the errors arising from manual operation and further diminishes the impact of operational experience on implant procedures.

## 4 Conclusion

We report a case of immediate implantation using an autonomous implant robot in a severe bone defect. Under the premise of ensuring the safety, the autonomous robot places the implants into the alveolar bone. The robot avoids thread exposure of the implant surface and ensures the initial stability of the implant. To the best of our knowledge, this clinical case is the first to report the feasibility and precision of immediate implantation in anterior teeth site with periapical cyst removal, performed by an autonomous robotic surgical system. Autonomous robot systems have emerged as a potential solution to tooth defects and are expected to become a mainstream medical technology in the near future. However, additional clinical trials are needed to verify the reliability of the system.

## Data availability statement

The original contributions presented in the study are included in the article/supplementary material, further inquiries can be directed to the corresponding author.

## Ethics statement

Written informed consent was obtained from the individual(s) for the publication of any potentially identifiable images or data included in this article.

## Author contributions

XL: Conceptualization, Investigation, Project administration, Software, Writing – original draft, Writing – review & editing. HL: Conceptualization, Data curation, Writing – review & editing. MC: Data curation, Investigation, Methodology, Software, Writing – review & editing. SC: Conceptualization, Data curation, Writing – review & editing. KJ: Data curation, Investigation, Methodology, Software, Writing – review & editing. SQ: Data curation, Investigation, Writing – review & editing. LZ: Data curation, Investigation, Writing – review & editing. YZ: Data curation, Funding acquisition, Investigation, Software, Writing – review & editing.
